# Amino Acid Usage Is Asymmetrically Biased in AT- and GC-Rich Microbial Genomes

**DOI:** 10.1371/journal.pone.0069878

**Published:** 2013-07-26

**Authors:** Jon Bohlin, Ola Brynildsrud, Tammi Vesth, Eystein Skjerve, David W. Ussery

**Affiliations:** 1 Centre for Epidemiology and Biostatistics, Department of Food Safety and Infection Biology, Norwegian School of Veterinary Science, Oslo, Norway; 2 Centre for Biological Sequence Analysis, Department of Systems Biology, Technical University of Denmark, Lyngby, Denmark; Tel Aviv University, Israel

## Abstract

**Introduction:**

Genomic base composition ranges from less than 25% AT to more than 85% AT in prokaryotes. Since only a small fraction of prokaryotic genomes is not protein coding even a minor change in genomic base composition will induce profound protein changes. We examined how amino acid and codon frequencies were distributed in over 2000 microbial genomes and how these distributions were affected by base compositional changes. In addition, we wanted to know how genome-wide amino acid usage was biased in the different genomes and how changes to base composition and mutations affected this bias. To carry this out, we used a Generalized Additive Mixed-effects Model (GAMM) to explore non-linear associations and strong data dependences in closely related microbes; principal component analysis (PCA) was used to examine genomic amino acid- and codon frequencies, while the concept of relative entropy was used to analyze genomic mutation rates.

**Results:**

We found that genomic amino acid frequencies carried a stronger phylogenetic signal than codon frequencies, but that this signal was weak compared to that of genomic %AT. Further, in contrast to codon usage bias (CUB), amino acid usage bias (AAUB) was differently distributed in AT- and GC-rich genomes in the sense that AT-rich genomes did not prefer specific amino acids over others to the same extent as GC-rich genomes. AAUB was also associated with relative entropy; genomes with low AAUB contained more random mutations as a consequence of relaxed purifying selection than genomes with higher AAUB.

**Conclusion:**

Genomic base composition has a substantial effect on both amino acid- and codon frequencies in bacterial genomes. While phylogeny influenced amino acid usage more in GC-rich genomes, AT-content was driving amino acid usage in AT-rich genomes. We found the GAMM model to be an excellent tool to analyze the genomic data used in this study.

## Introduction

The base composition in prokaryotes sampled from GenBank varies from 25% to 86% AT (*Anaeromyxobacter dehalogenans* strain 2CP-C and *Candidatus* Zinderia insecticola strain CARI, respectively). In some bacteria as much as 1% of the genomic base composition can change due to mutations in as little as 1400 years [1] resulting in a considerable impact on protein evolution due to the high fraction of protein coding DNA in microbial genomes [2]. Therefore, one of the central questions in prokaryotic evolution is what drives the direction of these mutations [3]? More precisely, is there a mutational bias towards AT-richness or GC-richness? If so, how does phylogenetic ‘inertia’ affect the mutational direction and to what degree are environmental factors responsible? How is all this affecting protein evolution? Laboratory experiments, statistical- and bioinformatical methods suggest that mutation towards AT-richness in prokaryotes may be due to loss of certain repair genes [4] and/or lack of selective constraints on the organisms usually termed collectively as selective pressures [5]–[7]. However, it has been more complicated to resolve what drives mutation in the direction towards GC-richness. Several findings indicate that microbial genomes become more GC-rich because they are subjected to stronger selective pressures [8], [9]. Gene conversion may result in GC-enriched genes which have been found to have elevated expression rates resulting in increased fitness (the ratio of viable offspring over total offspring for a particular species) [1]. GC-rich genomes have also been found to consist of less ‘random’ oligonucleotide frequency distributions [10]–[12]. In the present work we explore how these mutational biases are associated with both genomic amino acid usage bias and codon usage bias, which we define as the over-expression, or under-expression, of one or several specific amino acids or codons over others (hence, not to be confused with codon adaption indexes such as CAI [13]). We term these measures Amino Acid Usage Bias (AAUB) and Codon Usage Bias (CUB) respectively, and both are identically calculated using the empirical standard deviation on all amino acid- and codon (trinucleotide) frequencies for each genome. High values of AAUB (or CUB) is interpreted as one or more amino acids (or codons) being preferred (or avoided) over the remaining, while low values of AAUB (or CUB) is understood as a more balanced genomic amino acid (or codon) preference. In addition, we discuss possible influences on amino acid- and codon-usage from purifying selection, random mutations and selective pressures in general using the concept of relative entropy [11]. This was carried out by first downloading 2032 microbial genomes from GenBank (See Table S1) (http://www.ncbi.nlm.nih.gov/genome/) and then analyzing both amino acid- and codon frequencies using principal component analysis (PCA). Furthermore, we applied a Generalized Additive Mixed-effects Model (GAMM) [14], [15] to analyze explanatory variables such as genomic %AT, genome size, relative entropy, AAUB and CUB, many of which exhibiting non-linear trends as well as hierarchical structures of dependency ranging from strong within species to weak within phyla.

## Results

### Amino Acid Usage in Prokaryotes

**Figure 1:**
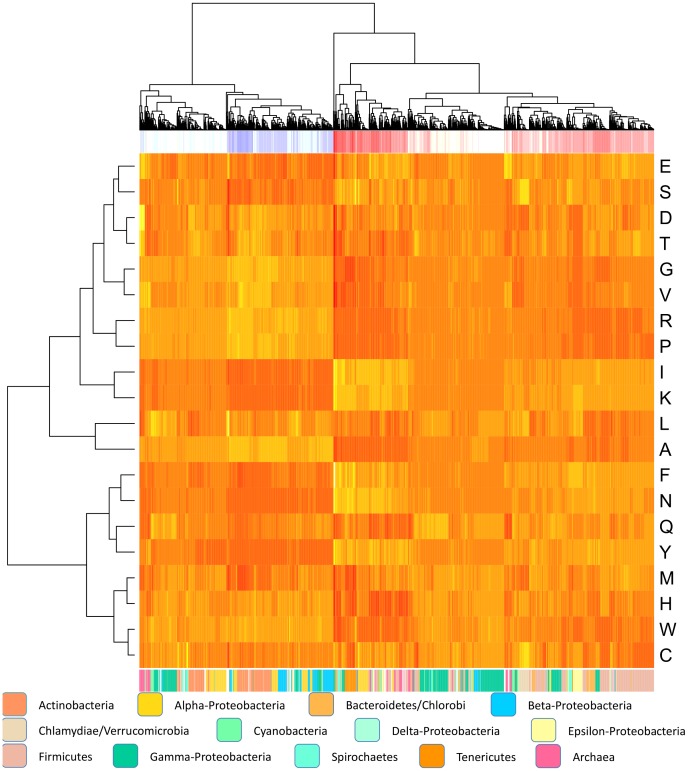
Heatmap of amino acid usage. The heatmap shows amino acid frequencies taken from 2032 bacterial genomes. Light colors represent higher frequencies while darker colors represent lower frequencies. The red and blue colors on the top bar represent GC content, where dark red and blue indicates AT- and GC-rich genomes, respectively. Genomes having GC/AT content close to 50% are represented by lighter grey colors. The bottom bar shows colors designating each genome’s phylum, which are detailed in the figure.

**Figure 2:**
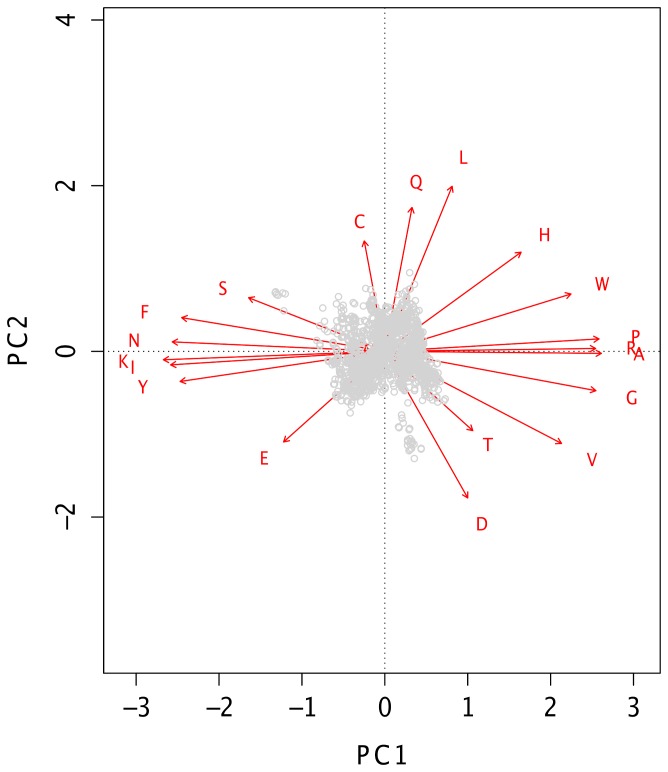
Principal component analysis plot. The plot shows two principal components resulting from a principal component analysis performed on the amino acid frequencies taken from 2032 bacterial genomes. The first principal component (PC1) was strongly associated with genomic %AT (decreasing left to right), while the second principal component (PC2) was associated with phyla.

Whole genome amino acid frequencies were calculated from the 2032 microbial genomes downloaded from NCBI GenBank. These amino acid frequencies were grouped using hierarchical, complete linkage clustering with Euclidean distance. The outcome of the cluster analysis can be observed from the heatmap in Figure 1, where the amino acid frequencies are colored with respect to occurrence; dark color - low frequency, light color - high frequency. We see from Figure 1 that amino acid usage is strongly linked with genomic %AT. A corresponding principal component analysis (PCA) carried out on the amino acid frequencies (see Figure 2) revealed that the first component explained over 80% of the variation in the data, indicating substantial similarity in amino acid usage between prokaryotes. A regression analysis between the first principal component and genomic %AT revealed an association of *R^2^ = 0.9* (*p<0.001*), while a regression analysis between the second principal component and phyla resulted in an association of *R^2^ = 0.74 (p<0.001)*. Hence, while the first principal component to a large extent described genomic %AT, the second component described phylogenetic influence. From the heatmap clustering and PCA we found that Isoleucine (I), Lysine (K), Phenylalanine (F), Asparagine (N), Tyrosine (Y), and to a lesser degree Serine (S) and Glutamic acid (E) were the most over-represented amino acids in AT-rich genomes (first principal component). Of these I, F and Y are hydrophobic, while K and E are positively and negatively charged, respectively, and S is uncharged. Lysine (K) was found to be the most over-represented amino acid in AT-rich genomes using PCA. In GC-rich prokaryotes we found that Glycine (G), Valine (V), Arginine (R), Proline (P), Alanine (A), and, to a lesser extent, Threonine (T), Histidine (H) and Tryptophan (W) were the most over-represented amino acids. Of these, V, A, W are hydrophobic, T is uncharged, and R and H are positively charged. The PCA analysis indicated that Alanine (A) was the most over-represented and characteristic amino acid for GC-rich bacteria. Cysteine (C), Leucine (L), Methionine (M), Aspartic acid (D) and Glutamine (Q), were found to be more evenly distributed in both AT- and GC-rich bacteria, while C, Q, L and D tended more towards the second principal component (phylogenic influence), indicating that these amino acids are more preferred by certain phylogenetic groups than others (see Figure 1). L and M (not visible, placed in the middle of both principal components) are hydrophobic, Q is uncharged, while D has negative charge and appears to be slightly more over-represented in GC-rich than in AT-rich genomes.

### Codon Usage in Prokaryotes

**Figure 3:**
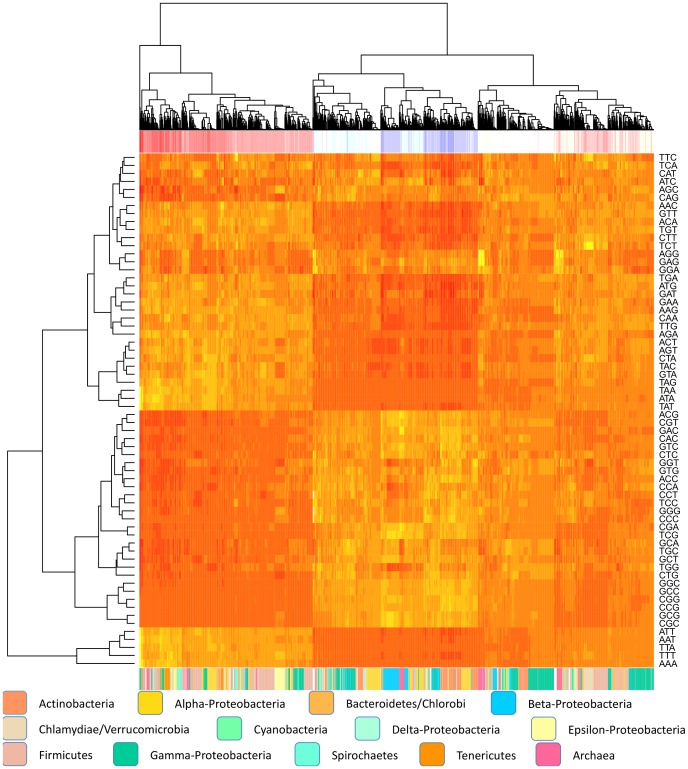
Heatmap of codon usage. The heatmap shows codon frequencies from 2032 bacterial genomes. Light colors represent higher frequencies while darker colors represent lower frequencies. The red and blue colors on the top bar indicate GC content, where dark red and blue represents AT- and GC-rich genomes, respectively. Genomes having GC/AT content close to 50% are represented by lighter grey colors on the top bar. The bottom bar shows colors indicating each genome’s phylum, which are described in the figure.

Figure 3 shows that there is a strong association between genomic codon frequencies and AT content. This is also supported by regression analysis fitted with genomic %AT for all prokaryotes as the response variable and all 64 codon frequencies as predictor variables. A positive linear association was found between genomic %AT (*p<0.001, R^2^ = 0.97*) and the following codons: AAA (Lysine), AAG (Lysine), AAT (Asparagine), AGA (Arginine), ATT (Isoleucine), GGG (Glycine), GCT (Alanine), GTT (Valine), CGA (Arginine), CCC (Proline), TCA (Serine), TCC (Serine), TCT (Serine), TTT (Phenylalanine), all *p<0.05*. A negative association was only found for the codon CTC (Leucine). PCA performed on the codon frequencies (See Figure S1) revealed that 20 components described 99% of the variation, one of which explained 80%. Similar to the PCA carried out for amino acids the first principal component exhibited a strong association with genomic %AT (*R^2^ = 0.96, p<0.001*), while the second principal component was associated with phylogeny (*R^2^ = 0.35, p<0.001*), although considerably less than what was observed for the amino acid based PCA (*R^2^ = 0.74*).

### Relative Entropy as a Measure of Cumulated Mutations

The concept of relative entropy is a cornerstone in information theory where it designates the divergence of one quantity measured against another [11], [16]. We use relative entropy to assess the information potential of codon (trinucleotide) frequencies. Since we are interested in the information potential of genomic codon frequencies we calculate the distance between observed codon frequencies and estimated codon frequencies with the Kullback-Leibler divergence (KL) (see Material and Methods section). The estimated codon frequencies are calculated using genomic nucleotide frequencies and represent therefore what we would expect if the neighboring nucleotides in codons were completely independent and determined only by average genomic %AT. Increased KL divergence denotes bias and is less likely to happen by chance as opposed to decreased KL divergence, which is more likely to happen by chance. Somewhat simplified and assuming mutations are random, KL can be thought of as a measure of genomic mutations accumulated over generations and time. We interpret KL to be a measure that is inversely proportional to the amount of acquired genomic mutations in the sense that decreasing KL is interpreted as increased rates of accumulated mutations under relaxed purifying selection. Since purifying selection removes deleterious mutations [17] it is reasonable to assume that species having been subjected to strong purifying selection have higher KL than species who have not. However, since loss of specific DNA repair genes like mutM and mutY have been associated both with increased mutations [4] and higher levels of genomic %AT, directly equating the KL measure with selective pressure may be misleading. It has been argued that the presence of specific tRNA genes may exert decisive influence on codon usage, and therefore also KL as discussed here, but this is controversial [18], [19].

### The Regression Models

To examine differences between relative entropy as measured using KL, AAUB, CUB and genomic %AT, we fitted several regression models. Since many of the above-mentioned factors were connected in a non-linear way and closely related organisms tend to have many similar properties causing cluster effects standard regression models could not be used. For example, some organisms are of more medical, commercial and scientific interest than others. These organisms, and their closely related species and strains, tend to be sequenced in larger numbers than organisms of lesser interest and statistical analysis involving such organisms may result in inaccurate regression models due to hierarchical clustering effects. For instance, the model organism *Escherichia coli*, had at the time of writing (October 2012) 54 fully assembled strains available at Genbank (not including 8 *Shigellas*), while another model organism, *Bacillus subtilis*, only had five strains available. Further, taxonomic inference between different strains and species can also be challenging; while the α-*Proteobacterial* genus *Brucella* consist of similar species [20], the *Cyanobacterial* species *Prochlorococcus marinus* vary greatly at the strain level [21]. In addition, although bacterial phyla like the Gram-positive *Firmicutes* and *Actinobacteria* are predominantly AT- and GC-rich, respectively, others like α- and γ-*Proteobacteria* contain species with a wide range of AT/GC-richness. Hence, since standard regression models assume somewhat independent observations, similar genomic properties within species, genera and phyla as described above can induce bias into the models, resulting in erroneous conclusions. A class of regression models collectively termed mixed-effects models [22] can, however, account for variance differences between groups such as species, genus or phylum. Since groups having a hierarchical structure can also be modeled using mixed effects regression we considered species, genus and phylum, respectively as independent levels in a hierarchical structure assuming variance to be similar within but different between levels. Similar levels of genomic %AT within species, genus and phylum, respectively were modeled as a random slope effect thereby accounting for progressive differences within each level. In other words, we assume that AT content is more similar within species, then genera and finally phyla. One class of regression models, called Generalized Additive Mixed-effects Models (GAMM) [14], [15], can handle non-linear associations and mixed effects modeling, and below we demonstrate the use of such models to examine associations between AAUB, CUB, genomic %AT and relative entropy. The regression models goodness-of-fit were assessed using Akaike’s Information Criterion (AIC) [23].

### The Link between Amino Acid and Codon Usage Bias

**Figure 4:**
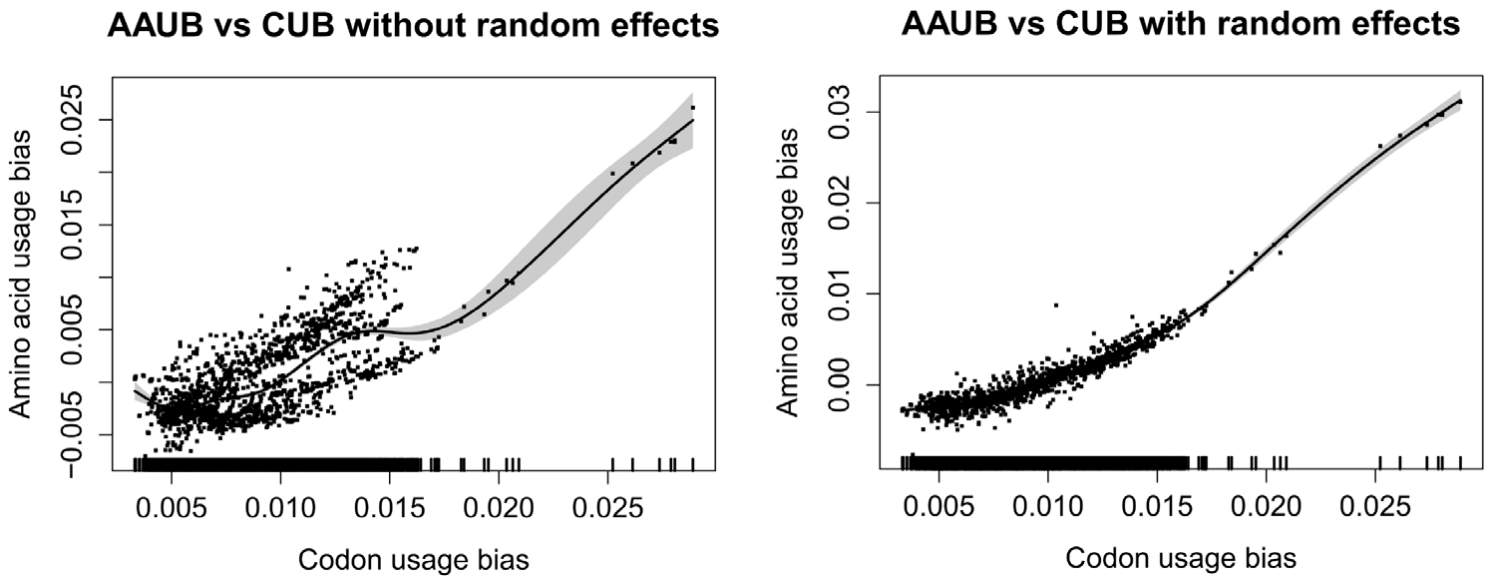
Amino acid usage bias versus codon usage bias. The Figure shows a GAM regression with amino acid usage bias on the y-axis (AAUB) as response regressed against the smooth of codon usage bias (CUB) on the x-axis. The dots represent the residuals together with the smoothed regression line. Both left- and right panels represent the same model, but the right panel is based on a GAMM model where strain, genus and phylum, with respect to AT content, are included as hierarchical random effects.

**Figure 5:**
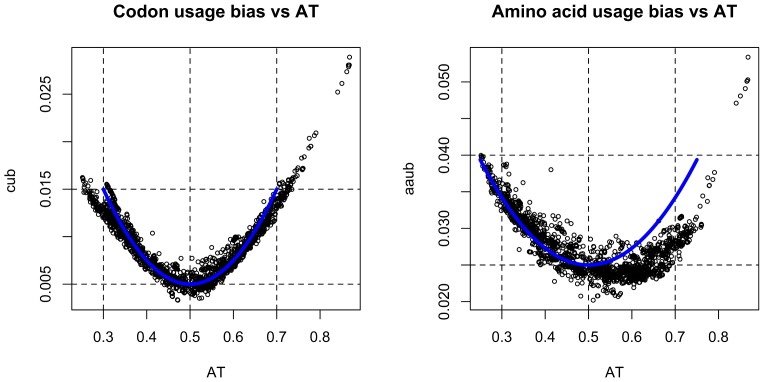
Codon and amino acid usage bias versus genomic %AT. The panels show codon and amino acid usage bias (vertical axis, left and right panel, respectively) plotted against genomic fraction of AT (horizontal axis) for 2032 genomes. The blue line shows what would be expected if the codon and amino acid usage bias were perfectly symmetrical for AT and GC-rich genomes.

We examined the link between codon frequency bias (CUB) and amino acid frequency bias (AAUB) for 2032 genomes (2152 chromosomes) with all plasmids removed. Figure 4 shows a GAMM regression with AAUB for each chromosome as the response and CUB as the only predictor, modeled using a smoothing spline. The left panel shows that there are considerable clustering effects due to greater similarity between phylogenetically close organisms. The right panel is the same regression model but with phylum, genus and strain added as hierarchical random effects with respect to AT content. It can be seen that the non-specified cluster effects have been reduced considerably. Furthermore, we found a clear, but weakly non-linear, association between AAUB and CUB. The non-linearity of the association indicates that there are fundamental differences between AAUB and CUB. Indeed, Figure 5 shows that AAUB (right panel) is more asymmetrically distributed with respect to genomic %AT as compared with CUB (left panel), which is more evenly distributed across AT- and GC- rich genomes. The marked trailing genomes that can be seen from Figure 5 are all strains of the insect symbiont *Candidatus* Carsonella ruddii, an organism known to have one of the smallest prokaryotic genomes (∼160 kb), and *Candidatus* Zinderia insecticola (also an insect symbiont) which has a slightly larger genome than *Candidatus* Carsonella ruddii and marginally higher AT content (86.5% AT compared to 86% AT for *Candidatus* Carsonella). An additional outlying genome can also be observed; the genome belongs to another insect-symbiont, *Candidatus* Hodgkinia cicadicola, which is a 58.4% GC α-*Proteobacterium* with the smallest bacterial genome known to date (∼144 kb). To examine which phylum had the highest and lowest AAUB we performed a standard ANOVA/linear regression type analysis which revealed that for bacterial phyla containing more than 20 species, *Bacteroides* (93 species) and *Firmicutes* (424 species) had the lowest and the second lowest average AAUB, respectively, while *Actinobacteria* (214 species) and β-*Proteobacteria* (169 species) had the respectively highest and second highest AAUB. The archaeal phylum *Halobacteriales* (21 species) had an average AAUB second only to the bacterial *Actinobacteriales* (214 species).

### Relative Entropy, Amino Acid Usage Bias and Genomic %AT

**Figure 6:**
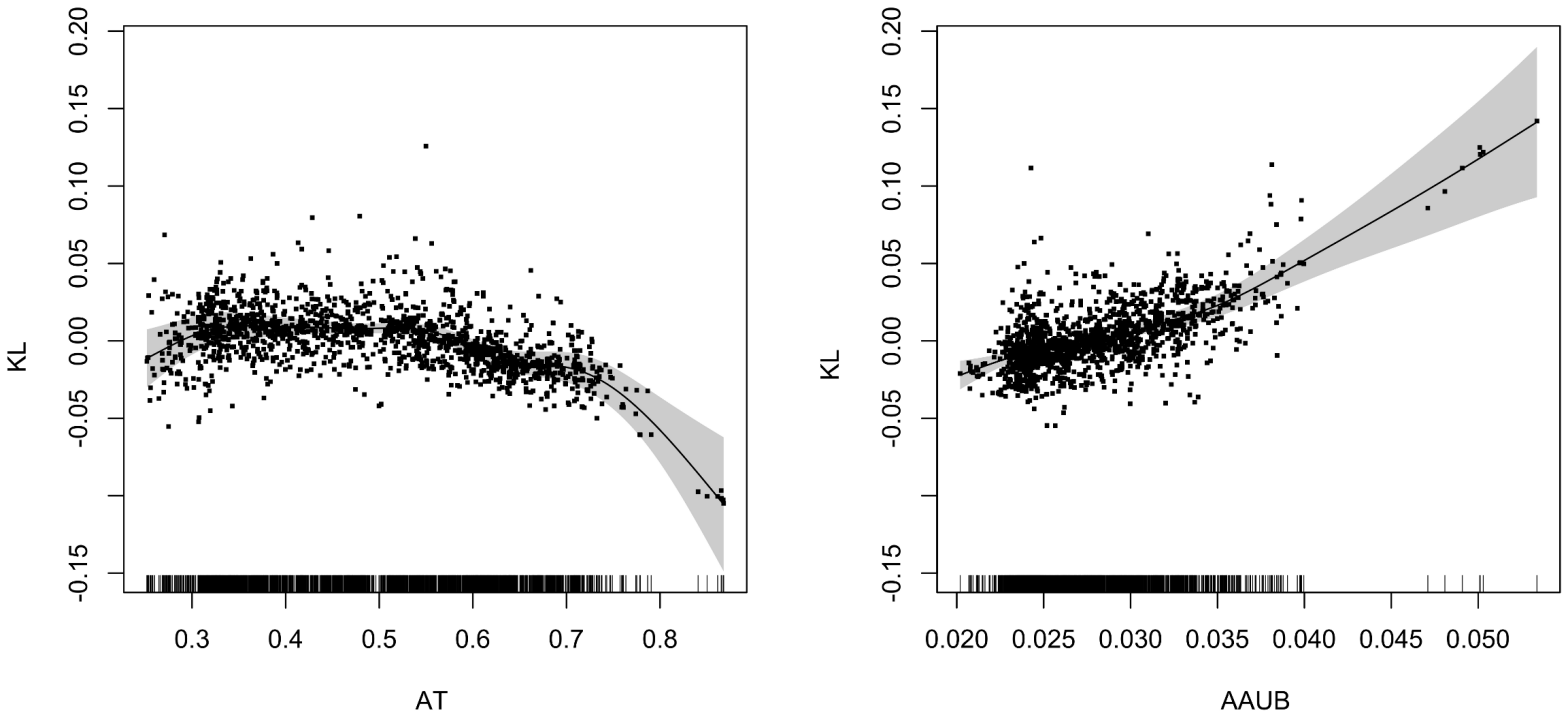
GAMM regression model of KL against AT content and AAUB. The panels show a GAMM regression model with relative entropy (KL) as response with genomic %AT and amino acid usage bias as predictors (left and right panels, respectively). Strain, genus and phylum have additionally been included as random effects with respect to genomic %AT. The dots represent the model residuals with respect to each predictor (AT content and AAUB) together with the spline estimated regression line. The shaded area surrounding the regression line indicates an interval of two standard errors.

To examine KL in all assembled prokaryotes we fitted a GAMM regression model with KL as response and both genomic %AT and AAUB as predictors, with phylum, genus and species as hierarchical random effects and AT content as a random slope effect. Figure 6 show the best model with AT content modeled using a smoothing spline and AAUB as a linear effect with the hierarchical random effects included. A considerable improvement in goodness-of-fit was observed from the AIC statistic with the inclusion of hierarchical random effects as compared to the model without random effects (AIC = −11697 with random effects compared to AIC = −9460 without). KL was found to decrease with increasing AT content, meaning that the genomic codon frequencies become more randomly distributed as genomes become progressively more AT rich. KL was found to increase significantly (*p<0.001*) with respect to AAUB, meaning that relative entropy and amino acid usage bias are positively associated such that genomic amino acid usage becomes more biased with increasing KL. A gross simplification and generalization of these results, for illustrative purposes, would be that AT-rich genomes have a more random base composition, due to accumulated mutations under relaxed purifying selection, than GC-rich genomes, *i.e.* AT-rich genomes contain more “noise” while GC-rich contain more “signal”. This may be due to GC-rich genomes having, on average, been subjected to stronger purifying selection than AT-rich genomes. Finally, it should be noted that GAM(M) regression carries out a back-fitting procedure implying that models are progressively fitted using all predictors as long as optimizations are possible. This procedure includes transforming the response, which is what is seen for AAUB and genomic %AT regressed against KL in Figure 6.

## Discussion

### Codon and Amino Acid Frequencies in Microbial Genomes

The principal component analysis applied to whole genome amino acid- and codon frequencies on all 2032 genomes indicated that the first two components were strongly associated with genomic %AT and phyla. While the association between genomic %AT and the first principal component was similar for both amino acid- and codon frequencies (R^2^ = 0.9 and R^2^ = 0.96, respectively), the association between the second principal component and microbial phyla differed considerably. Indeed, phyla explained 74% of the observed variance from the second principal component of the amino acid based PCA, but only 35% of the codon based PCA. Hence, phyla explained approximately 39% more of the variance in the amino acid frequencies than in the codon frequencies, indicating that genome wide amino acid frequencies contain a substantially stronger phylogenetic signal than codon frequencies. An asymmetric distribution of genome wide amino acid frequencies can also be observed from the PCA in Figure 2 where the amino acids typically found in AT-rich microbes (left part of horizontal axis in Figure 2) center more around the horizontal, genomic %AT associated principal component (PC1), while the amino acids predominantly found in GC-rich microbes (right part of horizontal axis in Figure 2) are visibly more distributed along the vertical principal component (PC2) associated with phylogeny. The PCA plot thus indicates that phylogeny is more strongly influencing amino acid usage in GC-rich microbes than in AT-rich and that AT content is influencing amino acid usage more in AT-rich than in GC-rich microbes.

### AAUB is differently Distributed in AT- and GC-rich Microbial Genomes

The observed almost linear relationship between CUB and AAUB as observed in Figure 4 was expected, since codons code for amino acids (albeit in a degenerative manner). With respect to base content, we see from Figure 5 that AAUB differs between AT- and GC-rich genomes. This means that the strongest bias in amino acid usage is found in species with low AT content, which is supported by numerous studies [24]–[27]. There are now many indications that mutations in prokaryotes are generally AT-biased, meaning that in absence of selective pressure prokaryotic genomes (and possible eukaryotes) become more AT-rich [5]. It has also been noted that organisms with AT-rich genomes in general have less biased amino acid usage, due to lack of selective constraints [24] which is also supported by the decrease in AAUB we observe for AT-rich prokaryotes. Many AT-rich bacteria, from several phyla, are often obligate intracellular organisms living in small populations, harboring genomes that are seldom recombined and often lack repair genes [6]. Nevertheless, from our data, reduction in AAUB appears to be a general trend linked to base composition and, as noted above, in support of recent and similar work on the subject.

### Factors Influencing Base Composition

From a purely statistical point of view both AT- and GC-rich genomes have access to the same number, although different types, of codons and amino acids, but from Figure 5 it can be seen that there are substantial differences in amino acid usage bias between AT-rich and GC-rich organisms. As has been noted previously, there is an association between genomic %AT and the environment the organism is isolated from [28]–[30]. As mentioned above, endosymbionts and intracellular bacteria are predominantly AT-rich. GC-rich bacteria, on the other hand, are often found in more versatile environments, having larger genomes with more genes than AT-rich bacteria, ultimately reflecting different selective pressures [31]. This asymmetry between genomic %AT and protein evolution has been noted before; indeed hydrophobicity values linked to the secondary structures of proteins correlate with GC content [25], [32]. Moreover, proteins of GC-rich bacteria have been found to be less susceptible to misfolding, but more prone to unfolding [27]. AT content was also found to be linked with population size in the sense that AT-rich bacteria were living in small population sizes while GC-rich bacteria were usually of intermediary population size [33].

### Relative Entropy and Selective Pressures

The GAMM regression shown in Figure 6 demonstrates an association between AT content and AAUB on one side and relative entropy, as measured using the Kullback-Leibler divergence, on the other. Since the KL measure estimates codon frequencies using only genomic nucleotide frequencies, the estimated codons, which are compared against the observed codons, reflect base composition in the respective genome only, and assume complete independence between the neighboring nucleotides in each codon. Hence, we see from Figure 6 that the neighboring nucleotides in codons become progressively more independent as genomic %AT increases. The dependence of the neighboring nucleotides in codons has been readily asserted and measured [34], therefore we consider low relative entropy (KL) to indicate that the species’ genome contains more cumulated mutations, since low relative entropy indicates more random codon frequencies, something we would expect if the mutations would be completely independent and random. It should be noted that although relative entropy decreased with increasing AT content, even organisms with low KL divergence have far from random codon frequencies. Therefore, decrease in amino acid usage bias (*i.e.* a wider preference for amino acids) and increase in genomic %AT is more likely caused by mutations under relaxed purifying selection due to the genome-wide inherent randomness in the estimated codon frequencies. These statistical associations are not irrefutable truths but trends as can be seen from figures 5 and 6. The route leading less AT-rich free-living and/or facultative symbiotic bacteria to become more AT-rich obligate intracellular bacteria with small genomes in small populations has been inferred for many species from many different phyla [2], [6], [26], [35], [36]. Assuming that genome reduction may be a consequence of increased mutation rates we (GAMM) regressed KL against genome size, but found no association (*p∼0.144*). Recombination is rare between obligate intracellular bacteria, and most such organisms seem to get by with what is provided by the host [31]. Therefore genes that produce proteins that are not essential are eventually shed [26]. Small, AT-rich populations have proteins that are hydrophilic, but, as was noted above, hydrophobicity changes with genomic %AT. Going from AT-rich to GC-rich and larger populations, proteins from prokaryotes become progressively more hydrophobic which points to increased selective pressure [27]. A previous study conducted by some of us, found that KL estimated from the DNA sequences of plasmids and phages was, in accordance with microbes, associated with AT content, but exhibited on average significantly lower KL than microbes, with plasmids having the lowest KL of all [11]. We therefore speculate that KL differences may be maintained by the sum of selective pressures the organism has been subjected to over generations and manifested through genomic %AT and amino acid usage bias, since proteins are in fact communicating with the extracellular environment [25]. Put more simply, evolution of AT-rich bacterial genomes appears to have been progressively more left to chance and, conversely, bacterial genomes with high %GC appears to have been better conserved and more strongly subjected to purifying selection since genomic codon frequency estimations became progressively more inaccurate with increasing %GC. What forces could be responsible for the more biased codon frequencies in GC-rich bacteria cannot be stated with certainty, although repeated purging of deleterious mutations (purifying selection) may at least explain some of the observed increase in AAUB [17]. In addition, several other studies have reported that GC-rich bacteria are often found in soil [3], [37]; temperature and oxygen requirement may [38], [39] (or may not [38], [40]) have an impact on GC content, the availability of nitrogen [41], population size and hydrophobicity [27] and rate of gene expression [1] have all been associated with elevated levels of genomic %GC in microbes. Gene regulation has also been found to be more complex in increasingly larger genomes, also correlating with genomic %GC [42]. Reva and Tümmler [10] found a similar association between tetranucleotide frequencies and genomic %GC and suggested that it could be due to the increased energy required to stack and de-stack GC-rich sequences, which was also pointed out by Rocha and Danchin in a previous study [3].

### Conclusions

We found that amino acid usage was strongly associated with genomic %AT but that phylogeny appeared to exert a stronger influence in GC-rich microbes. Furthermore, our results indicate that whole genome based amino acid frequencies carried a substantially stronger phylogenetic signal than codon frequencies. An asymmetry in amino acid usage bias between AT- and GC-rich genomes was also detected and this asymmetry was found to be associated with relative entropy in the sense that relative entropy was found to increase with amino acid usage bias. Since closely related organisms tend to differ less with respect to base composition than more distantly related species, we propose the use of GAMM to circumvent the assumption of independence and linearity in standard regression analysis. The negative association found using GAMM regression between relative entropy and amino acid usage bias indicate that genomic %AT might be an indicator of the selective constrains. However, since the loss of specific repair genes increases the number of cumulated mutations and genomic %AT, regardless of the selective pressure the species has been subjected to, establishing a direct causative link with relative entropy is at this stage premature. Our findings also support that genomic %AT in microbes is not independently associated with the environment, but is additionally conditioned on phylogeny in the sense that large differences in genomic %AT between organisms living in similar habitats may be due to large genomic %AT differences in their respective ancestors [43]. Nevertheless, to establish a more firm causative relationship between selective pressures, base content and amino acid usage in microbes more research is needed.

## Materials and Methods

All 2032 genomes and corresponding open reading frames were downloaded from NCBI Genbank (http://www.ncbi.nlm.nih.gov/genome/) accessed October 2012 (see Table S1). All plasmids were removed. In-house scripts were used to estimate all factors discussed (AAUB, CUB, AT, size, KL, etc.) and are available upon request.

### Measurement of AAUB and CUB

Scripts were written to estimate amino acid frequencies from protein files and codon frequencies from DNA ORF files. Usage bias for both amino acids and codons were calculated as the empirical standard deviation of the resulting 20 and 64 frequencies, for each genome, respectively:
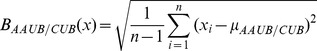
where *n = 20/64* depending on whether amino acid- or codon frequencies are used. *x_i_* and *μ* designates amino acid-/codon- frequency and mean frequency, respectively. The statistical distributions of both genomic codon- and amino acid frequencies were examined using distributional plots for several genomes (including the most AT- and GC-rich genomes) and all cases examined were found to be approximately normally/symmetrically distributed.

### Statistical Analyses

All statistical analyses were performed with the free statistical language R [44] (“http://www.R-project.org/”). Generalized additive mixed-effects regressions were performed using the packages “gamm4” and “lme4” [45], [46].

The codon- and amino acid frequency heatmaps were created using the “heatmap” command in R, which performed hierarchical clustering with “complete” linkage (focusing on the farthest neighbors for robustness) and “Euclidean” distance. PCA was carried out using amino acid- and codon frequencies and estimated with a correlation matrix using the “vegan” package [47]. Standard linear regression was carried out between the two first principal components and genomic %AT and phyla, respectively.

Codon usage versus AT content was examined using linear regression with genomic %AT for each genome *i* as the response (*y*) with each corresponding codon frequency (*x_1_*,…,*x_64_*, 64 “codons” in total) as predictors and the parameters *β_m_(0≤m≤64)*, (*m = 0* for intercept) to be estimated. *ε* is the normally distributed model error:
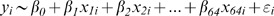



To assess the association between AAUB and CUB we used a generalized additive model with AAUB for each genome *i* as the response (*y*) and the corresponding CUB (*x*) as the predictor modeled using a smoothing spline (*s(.)*):
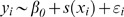
(1)


Again *ε* designates the model error term and *β_0_* the intercept. In addition, a hierarchical mixed effects model was made (from top to bottom) with strain/genus/phylum (*j,k,l,* respectively) as random hierarchical effects (**Z**), all with respect to genomic %AT (*w*), making the model in practice a hierarchical random slope model:
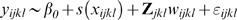
(2)


The difference between models (1) and (2) can be seen in Figure 4. The comparison of AAUB between phyla was carried out using a simple linear regression model with AAUB for each genome *i* as response (*y*) and a categorical predictor *x_j_*, consisting of *j = 34* different phylogenetic groups and corresponding vector of estimated parameters ***β*** *_1_*, with error *ε* and intercept *β_0_*:
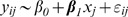



Relative entropy was measured using the Kullback-Leibler divergence of genomic codon frequencies versus estimated codon frequencies [11], *i.e. F_i_(XYZ)∼F_i_(X)F_i_(Y)F_i_(Z)*, *F_i_* being a frequency function for a genome *i* and *X*, *Y* and *Z* respective nucleotides {A, G, C, T} of a codon *XYZ*:
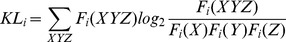



The sum is hence taken over all possible codon frequencies for each genome *i*.

To estimate the association between KL, AAUB and AT content we fitted a GAMM model similar to equation (2) above, but with KL for each genome *i* as response and predictors AAUB (*x_1_*) and genomic %AT (*x_2_*). Again we added strain/genus and phylum with respect to genomic %AT as hierarchical random slope effects:
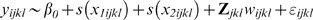



The goodness-of-fit of the GAMM based models were assessed using the Akaike Information Criterion (AIC) [23].

## Supporting Information

Figure S1 **PCA plot of codon frequencies.** The plot shows two principal components resulting from a principal component analysis performed on the codon frequencies taken from 2032 bacterial genomes. The first principal component (PC1) was strongly associated with genomic %AT (decreasing left to right), while the second principal component (PC2) was associated with phyla.
(PDF)

Table S1 **Dataset.** The dataset in Excel format used in the article. The file includes NCBI accession numbers for all DNA sequences used.
(XLS)
