# Supplementary figures and images for: Amino Acid Usage Is Asymmetrically Biased in AT- and GC-Rich Microbial Genomes
Source: PLoS One. 2013 Jul 26;8(7):e69878. doi: 10.1371/journal.pone.0069878 (PMC3724673; doi:10.1371/journal.pone.0069878)

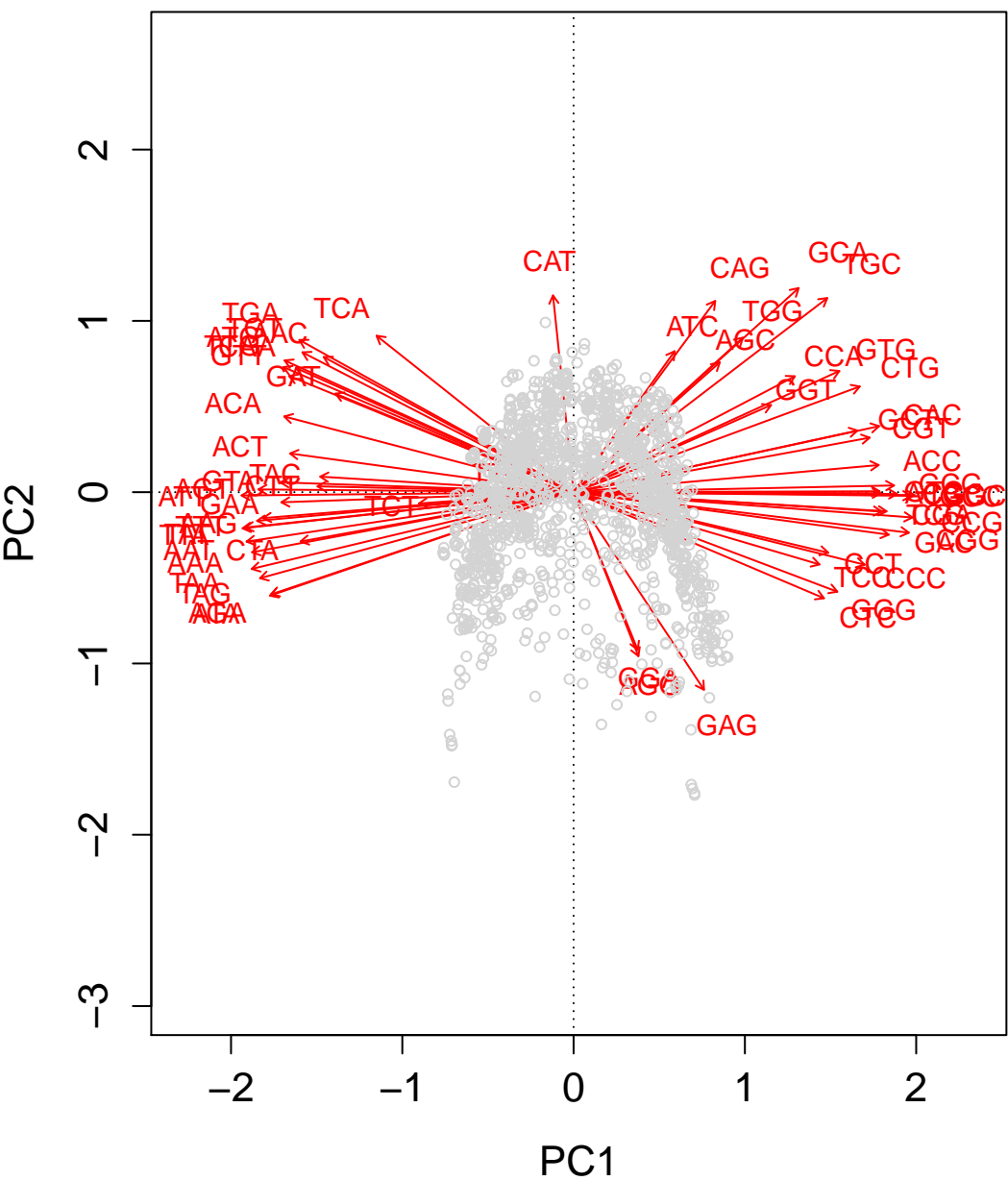

Supplement: Figure S1 — PCA plot of codon frequencies. The plot shows two principal components resulting from a principal component analysis performed on the codon frequencies taken from 2032 bacterial genomes. The first principal component (PC1) was strongly associated with genomic %AT (decreasing left to right), while the second principal component (PC2) was associated with phyla. (PDF) [file pone.0069878.s001.pdf]
